# Careers of young Polish chemists

**DOI:** 10.1007/s11192-014-1461-x

**Published:** 2014-10-31

**Authors:** Marek Kosmulski

**Affiliations:** Lublin University of Technology, Lublin, Poland

**Keywords:** Doctorate, Master’s degree, Doctoral studies, Gender, Age, Academic inbreeding

## Abstract

Typical young Polish scientist is an alumnus of doctoral studies at the same university and department where he/she completed his/her Master degree. The career is continued by receiving a habilitation at the same university and department. Then a holder of habilitation is promoted to a tenured position at the same university and department. Detailed analysis of scientific careers of 154 recent Ph.D. recipients and of 16 habilitation candidates in chemistry from University of Warsaw is presented. More than 96 % of the Ph.D. theses were results of doctoral studies. A typical doctor is Polish citizen (>98 %), alumnus/alumna of the University of Warsaw (>85 %), holder of Master degree in chemistry (88 %) who joined the Ph.D. program at the same university directly after having completed his/her Master degree, and completed the Ph.D. program 5.5 years after completion of Master degree. A fraction of recent female Ph.D. recipients in chemistry (61 %) is very high as compared with the corresponding fractions in other countries (e.g., USA), but it is still substantially lower than the fraction of female Master degree recipients. In recent habilitation candidates, the female ratio is 50 %, thus relative male dominance is observed at higher levels. At least one-third of the recent Ph.D. recipients were employed by the same university, where they received their Ph.D., while the fraction of the recent Ph.D. recipients employed by other universities in Poland was below 5 %. High degree of academic inbreeding is due to the legal system in Poland, which (nominally) is designed to prevent academic inbreeding, but the regulations can be easily circumvented. Over 10 % of the recent Ph.D. recipients found post-doctoral positions abroad, chiefly in EU countries and in the USA.

## Introduction

Different aspects of scientific careers in the USA and in Western Europe have been extensively discussed. Kaulisch and Enders (2005) emphasize the differences between a chair model, which is represented by academic careers in Germany and department-college model, which is represented by academic careers in the USA. In Germany there are relatively few professorial positions, and advancement to such a position is considered as a big jump in terms of job security, prestige and resources. In contrast the status of academic staff in the USA is less dependent of their positions (junior staff vs. full professor), and it is strongly influenced by current publication record and successful grant applications. The appointment to a tenured position in Germany comes relatively late (age over 40), and it follows a series of short-term appointments. In the USA the tenure track positions are available for recent Ph.D. recipients. The other essential difference is that German regulations do not allow advancement of junior scientists to professorial positions within the same organization (university). Thus, institutional mobility of academic staff is enforced by law. In the USA there is an “usual expectation” that junior researchers change universities after completion of their Master degree, and then after completion of their Ph.D., but an academic career within the same institution is allowed by law. Nevertheless, the fraction of faculty members who graduated from the same school in the USA is typically <20 % (Smyth and Mishra 2014). The level of academic inbreeding in top American universities (Harvard, Yale) is substantially higher than in less prestigious universities. Unlike Germany and USA, the institutional mobility in Sweden and Norway is low, that is, the academics stay at the same institution over their entire careers (Musselin 2004). The academic inbreeding has been considered as a possible factor retarding the development of science (Anonymous 1998; Inanc and Tuncer 2011).

Musselin (2002) discussed the differences in faculty recruitment between French and German universities. The recruitment is strongly affected by legal regulations which are specific for given country, and which are not encountered in most other countries. For examples the highest professorial positions in France are only available for candidates who have passed a special state exam (*agrégation du secondaire*). Also the distinction awarded for the doctorate plays a decisive role in academic careers in France. There are three levels of distinction (*honorable*, *très honorable*, *très honorable avec félicitations du jury*), and only the candidates who received at least the second level of distinction have a chance for a professorial position. In Germany, only candidates with habilitation are considered for professorial positions. The above criteria are not met by most hypothetical foreign candidates, especially by candidates from countries, where such legal regulations do not exist.

The difference between French and German systems results in a substantial difference in the age profile of holders of tenured positions: typical age of access to a tenured position is about 33 in France, and 42 in Germany (Musselin 2004).

In contrast to the Western countries, the scientific careers of scientists in the countries of the former Soviet Bloc are not that well documented. The present paper is an attempt to fill this gap. Scientific careers of young Polish chemists will be discussed in detail, but the trends found in the present study are probably representative for other scientific disciplines, as well.

The general overviews on doctorate recipients in Poland are readily available (doctorates by sex and by discipline) in statistical yearbooks (Statistical Yearbook of the Republic of Poland 2013). In contrast, the availability of specific data on individual doctorates is limited. There are numerous databases on individual doctorate recipients in Poland, e.g., (Nauka Polska 2014; POLON 2014), but they mainly cover the scientific aspects (title, scientific discipline) while personal and private information is concealed. It will be shown later in this paper that those databases are incomplete, and they often report erroneous data. Institutional and field-to-field migration of Polish scientists was studied using a large sample (Batorski et al. 2010). However, that study was based on the data extracted from Nauka Polska database, which is neither complete nor accurate, thus, the significance of the results is limited.

The present study is devoted to careers of young scientists in Poland, and it is based on data about their doctorates obtained from all possible sources including the documents deposited at Dean’s office of the Chemical Faculty of the University of Warsaw. The structure of the data collected was inspired by the Survey of Earned Doctorates (2012).

## Definitions

The terminology used in this paper refers to the country-specific educational system. In Poland the Ph.D. procedure is regulated by the state. A few details of the Ph.D. procedure have been changed in course of the studied period (2007–2014), but the main principles remain unchanged. In Poland, doctorate in the first (lowest) scientific degree. Complete Master’s degree (2nd cycle of higher education in the sense of Bologna Process) is a pre-requisite for Ph.D. candidates. Bachelor’s or Engineer’s degree (received after 1st cycle of higher education in the sense of Bologna Process) is not sufficient. Master’s degree is a professional degree (title) in Poland, and it is not a scientific degree. In this respect Poland is very different from USA, where only 63 % doctorate recipients in physical sciences were holders of Master’s degree (2012).

There is a limited number of doctorate granting institutions. A successful Ph.D. defense is subject to approval by a Faculty Council (or similar body). Not always is the Ph.D. approved (awarded) on the day of defense. Ph.D. holders may apply for higher degrees, that is, habilitation (Dr. Sci.), and professorship. The term “professor” in Poland may refer to a degree (title) on the one hand, and to a position on the other. Not all holders of professor degree have professorial positions and vice versa. The procedure leading to habilitation and to professor degree (title) is long, complex, and it underwent numerous changes over the recent few years. Only a holder of habilitation or professorship may act as an adviser of Ph.D. candidate or as a referee of doctoral thesis. Typically single holder of habilitation acts as an adviser. In international Ph.D. programs, there are two advisers (one from each university). Two Polish advisers are allowed, but such a possibility is seldom used.

Ph.D. degree is not required for a position of university teacher, and teachers who only hold Master’s degree are commonplace. However, only holders of Ph.D. may apply for higher positions in the academic hierarchy. The doctorate is subject to distinction (one level of distinction), and such a distinction is not formally required in faculty recruitment.

Doctoral students are participants of the 3rd cycle of higher education in the sense of Bologna Process. About 20 % of them receive modest scholarships. Only a few doctorate granting institutions offer doctoral studies.

Using the terminology from Musselin (2004) the selection system in Polish scientific institutions can be described as “up-or-out” system at early career stage (before habilitation) and “promotion” system after habilitation (the candidates who are not advanced to higher professorial positions do not have to leave their scientific institution).

## Data collection

Ph.D. theses in chemistry defended at the University of Warsaw since 2007 have been studied. In terms of publication and citation record, the Department of Chemistry of the University of Warsaw is the strongest chemical department in Poland. For example at least six top-h faculty members of Department of Chemistry of University of Warsaw have each a higher personal *h*-index than the overall *h*-index of University of Opole, or of Rzeszów University of Technology (both universities have chemical departments). Among 27 papers with Polish affiliation published over the period 2005–2014 in Chemical Reviews, which is one of the most prestigious chemical journals, as many as 5 (including 3 of 6 most-cited ones) were from University of Warsaw.

Therefore the scientific achievements of those newly promoted doctors are not necessarily representative for other scientific institutions in Poland. The data regarding the doctorate recipients (sex, citizenship, birthday), their former education (engineer degree; master degree: date, field, institution), and their doctorate (date, field, institution, adviser(s), referees) was extracted manually by inspection of relevant documents deposited at and issued by the Department of Chemistry of the University of Warsaw. Such documents are stored for limited time (5 years or so after Ph.D. defense) in the Dean’s office, and then transferred to the University’s central archive, which is a common practice in Polish universities, and those documents are not accessible to the general public. The information was redundant, that is, the same data (e.g., birthday of doctorate recipient) were often reported in more than one document.

The present method of data collection is different from that used in most published journal papers (Villaroya et al. 2008; Ardanuy et al. 2009), where the information on Ph.D. recipients and theses was extracted from databases of doctorates. The present method of data collection is different from Survey of Earned Doctorates (2012), which is based on data provided by the doctorate recipients. The present method does not assure completeness of data. However, all possible efforts have not detected any case of missing information.

The data collected at the Dean’s office was also compared with the data included in “Nauka Polska” (2014) and “POLON” (2014), which are Polish national databases of holders of scientific degrees. Those databases are accessible to general public (no username and password required). The information reported in “POLON” is limited to the name of the Ph.D.-holder, the scientific discipline, and current position (if employed in an academic institution). This information is often not sufficient to assure that the POLON-record and the data collected at Dean’s office refer to the same person, especially with common last names (possible homonymous Ph.D.-holders). The information reported in “Nauka Polska” is more detailed, e.g., it contains the name of doctorate-granting institution, the names of the adviser(s) and of referees. This information is sufficient to assure that the “Nauka Polska”-record and the data collected at Dean’s office refer to the same person. Discrepancies between the “Nauka Polska”-record and the data collected at Dean’s office were commonplace, and they referred to the date of doctorate, the role of particular professors in the Ph.D. procedure (adviser vs. referee), middle name of doctorate recipient, etc. “Nauka Polska”-records are based on survey form sent by Dean’s office to the database administrator. Therefore the present author believes that the information extracted from the original documents at the Dean’s office is the correct one in case of discrepancies. The information about 16 recent habilitation candidates was obtained by similar means.

The CVs of the doctorate advisers were based upon the aforementioned “Nauka Polska” and POLON databases (which provide more complete and accurate information about professors than about recent doctorate recipients), and on an official complete list of Master, Ph.D., habilitation, and professor degrees (titles) awarded by Department of Chemistry of the University of Warsaw till 2005 (Wielogórski 2005).

## Doctorate recipients

The analysis covers 154 records. The distribution of doctorates by year is presented in Table 1. On average, 21 doctorates per year were approved (SD 6) over the period 2007–2013.

**Table 1:** Doctorates by year

Year	Number
2007	29
2008	23
2009	23
2010	20
2011	13
2012	15
2013	29
2014	2

All 154 doctorate defenses were successfully defended and approved, that is, there was no case of unsuccessful defense, or of unapproved successful defense. The doctorates had two referees each, which is a minimum number required by law. Additional referee is allowed, but this option has not been used. The reviews were 100 % positive. This picture is typical for Polish (and not only Polish) universities, and it confirms the general opinion, that Ph.D. defense is a rite (Lariviere 2012). The information on doctorates and on doctorate recipients is summarized in Table 2.

**Table 2:** Characterization of doctorates and of doctorate recipients

Property	Total	(%)
Data on Ph.D. recipient		
Female	94	61
Polish citizen	151	98
Participant of doctoral studies	148	96
Previous professional career		
Employee of University of Warsaw	0	0
Employee of another university	1	1
Previous education		
Recipient of Master degree in chemistry	136	88
Recipient of Engineer degree	12	8
Alumnus of University of Warsaw	131	85
Alumnus of University of Warsaw, Dept. of Chemistry	126	82
Ph.D. details		
Ph.D. under 28	8	5
Ph.D. under 30	94	61
Ph.D. <5 years after Master	46	30
Ph.D. <7 years after Master	131	85
2 advisers from Poland	5	3
Additional adviser from abroad	6	4
Co-adviser	3	2
Coverage in Polon and Nauka Polska		
Correct and relevant information in Polon	92	60
Correct information in Nauka Polska	101	66
Correct information in Polon and in Nauka Polska	60	39
No record in Polon	53	34
No record in Nauka Polska	29	19
Further career (current or past position)		
University of Warsaw	53	–
Other university	7 (6 in Poland)	–
Polish Academy of Sciences, and other scientific institutes	12	–

The fraction of female Doctors was 61 % and it was much higher than the fraction of female Doctors in chemistry in the USA, which ranged from 32 to 38 % over the period 2002–2012 (2012). On the other hand, the fraction of female Doctors was lower than a fraction of female Masters of chemistry (74 %) promoted by Department of Chemistry of University of Warsaw between 2002 and 2005 (Wielogórski 2005). Therefore, the present results support the general opinion that the scientific hierarchy is male-dominated (Kretschmer and Kretschmer 2013).

Only two foreign citizens (in one case the citizenship could not be unequivocally stated) obtained a Ph.D. degree in chemistry from University of Warsaw in the studied period. Very low degree of internationalization is typical for Polish universities. In contrast only a half of the doctorates in physical sciences in the USA in 2005 was earned by US citizens or permanent US residents, and the other half was earned by foreigners (temporary visa holders), and the fraction of temporary visa holders among the newly promoted doctors was steadily increasing (2012). Over 96 % of the Ph.D. theses were results of doctoral studies, that is, the 3rd cycle of higher education in the sense of Bologna Process. Only one doctorate recipient held a position of university teacher, and no Ph.D. recipient was an employee of University of Warsaw in course of Ph.D. preparation. In this respect the careers of the recent doctorate recipients are very different from the careers of their advisers (professors), who worked on their doctorates as employees of University of Warsaw.

Most doctorate recipients (136) had Master degree in chemistry. The other recipients of doctorate earned their Master degrees in chemical technology (8), physics (2), biotechnology, environmental chemistry, environmental technology (2), clean technologies, biology (2) and mechanical engineering. Six recipients of doctorate earned a Master degree in chemistry and additionally a Master degree in another discipline. As many as 131 doctorate recipients had a Master degree from the University of Warsaw, and 126 of them had a Master degree from the Department of Chemistry of the University of Warsaw (doctorate granting institution). Thus the number of field-to-field migrations and the number of institutional migrations of scientists at Ph.D. level were both below 20 % of the total number of recent doctorate recipients.

The youngest doctorate recipient was 27 years and 3 months old on the day of Ph.D. approval, and 8 doctorate recipients (5 %) were below 28. The median age of the doctorate recipients was 29.6 years, and the average age was 30.3 years. The shortest period between Master’s degree and doctorate was 3 years and 9 months, 46 doctorate recipients (30 %) made it within less than 5 years, and 131 doctorate recipients (85 %) made it within less than 7 years. The median period between Master’s degree and doctorate was 5.5 years, and the average period was 6.1 years. These results indicate that the official length of doctoral studies in most Polish universities, which is 4 years, is unrealistic.

Thus a young Polish scientist (the generation born about 1980) had a chance for his/her first paid job in the age of about 30. This job is not a secure position, and it is subject to up-or-out system. A secure position depends on successful habilitation. In this aspect, Polish system is similar to the German system.

Table 2 indicates that data on substantial fraction of the actually received doctorates is missing or incorrect in the databases Polon and Nauka Polska. Therefore analyses based solely upon those databases are likely to produce incorrect results. There is no correlation between the coverage in Nauka Polska and the year of the doctorate, and moderate correlation between the coverage in Polon and the year of the doctorate (higher coverage of recent doctorates).

The documents deposited at and issued by the Dean’s office used in the present study refer to the status before or just after the Ph.D. graduation and they do not cover further professional careers of the doctorate recipients. In contrast, the databases Polon and Nauka Polska are steadily updated, and they are supposed to provide information on the current employment of the doctorate recipients. According to the databases (Table 2), a holder of Ph.D. from University of Warsaw has a good chance of having received a job at the same University, some chance of having received a job in a scientific institution other than university, and almost no chance of having received a job at an university other than his/her own Alma Mater in Poland. According to legal regulation in Poland, each position opening at a state university has to be publicly advertised and all applications (internal and external) have to be considered. Yet the faculty council is free to define the profile of the candidate. In order to circumvent the requirement of obligatory open competition the profile of the candidate can be so defined (e.g., in terms of very narrow specialization) that only one specific candidate meets all the criteria, and the open recruitment required by law becomes a fiction.

The publication records from WoS^®^ were used to identify those Ph.D. recipients who continued their scientific careers outside Poland. The Ph.D. recipients who were not employed by a scientific institution in Poland (according to Polon and Nauka Polska databases, cf. Table 2) or whose records were missing in both databases were examined for their publication activity since the second next year after completion of their Ph.D. It was assumed that the publications up to the year following the Ph.D. may be related to their doctorates. This method does not apply to very recent (2013 or 2014) Ph.D. recipients and to scientists whose last names are very popular (multiple homonymous doctors). Out of 55 analyzed records (Ph.D. recipients until 2012, whose employment at Polish scientific institutions was not reported in Polon or Nauka Polska databases, and whose last names are not very popular) only 26 have not shown any publication activity after completion of their Ph.D., 16 published scientific papers with Polish affiliation (chiefly University of Warsaw) and 13 published scientific papers with foreign affiliation (USA 6, EU 5, Japan 1, Saudi Arabia 1). This result indicates a substantial level of brain drain. Interestingly enough, there are very few Polish citizens among the foreigners who earned their Ph.D. in the USA (2012). Apparently Polish citizens prefer to earn their Ph.D. in Poland and then apply for post-doc positions in the USA rather than to earn their Ph.D. in the USA.

## Habilitations

The analysis was based on 16 records covering the period (date of definitive decision) 2012–2014. Two habilitations were approved in 2012, 12 habilitations were approved in 2013, and one habilitation was approved in 2014. The covered period is shorter than for doctorates (cf. Table 1) due to limited availability of documents. The characterization of habilitations and of habilitation candidates is presented in Table 3.

**Table 3:** Characterization of habilitations and of habilitation candidates

Property	Total	(%)
Data on habilitation applicant		
Female	8	50
Polish citizen	16	100
Previous professional career		
Employee of University of Warsaw	13	81
Employee of another university	3	19
Previous education		
Recipient of Master degree in chemistry	16	100
Recipient of Engineer degree	0	0
Alumnus of University of Warsaw	13	81
Alumnus of University of Warsaw, Dept. of Chemistry	13	81
Ph.D. details		
Ph.D. in chemistry	15	94
Ph.D. under 28	1	6
Ph.D. under 30	10	63
Ph.D. <5 years after Master	7	44
Ph.D. <7 years after Master	13	81
2 Advisers from Poland	0	0
Additional adviser from abroad	0	0
Habilitation details		
Successful in the first attempt	13	81
Successful after appeal	2	13
Failed	1 (male)	6
Habilitation under 40	5	33^a^
Habilitation under 45	11	73^a^
Habilitation <10 years after Ph.D.	2	13^a^
Habilitation <12 years after Ph.D.	7	47^a^
Coverage in Polon and Nauka Polska		
Correct and relevant information in Polon	12	75
Correct information in Nauka Polska	9	56
Correct information in Polon and in Nauka Polska	7	44
No record in Polon	2	13
No record in Nauka Polska	0	0

^a^Successful candidates

Unlike with doctorates which had rather ritual character the habilitation procedure is a real decision-making process. Negative reviews and even negative final decisions are commonplace. The fraction of female habilitation candidates was 50 % (53 % in successful candidates), which is lower than the fraction of female doctors (Table 2). This should be emphasized that the results presented in Table 3 refer not only to a higher level of scientific career, but also to different age group of scientists than the results presented in Table 2.

All candidates were Polish citizens, employees of Polish universities, and holders of Master degree in chemistry. Lack of interest of foreign candidates in Polish habilitation can be easily explained, namely there are relatively few countries, in which habilitation is useful as a scientific degree. All candidates but one (Ph.D. in chemical technology) received their Ph.D. is chemistry. 13 candidates (81 %) received both Master and Ph.D. degree in the Department of Chemistry of University of Warsaw. These results indicate low degree of institutional and field-to-field mobility of habilitation candidates.

The youngest successful candidate received her habilitation in the age of 37 years and 3 weeks, and the median and average age of the candidates were 42.2 and 43.5 years, respectively. The shortest period between doctorate and habilitation was 8 years and 5 weeks, and the median and average period between doctorate and habilitation were 12.1 and 12.6 years, respectively.

## Advisers and referees

Usually these was one adviser per one Ph.D. thesis with a few exceptions indicated in Table 2. Fifty-six Polish professors participated in the doctorates as advisers including 15 female professors. The fraction of female advisers (professors) who participated in the doctorates discussed in this paper is much lower than a fraction of female doctors. This should be emphasized that the above fraction of female scientists refers not only to a higher level of scientific career, but also to different age group of scientists than the fractions presented in Tables 2 and 3.

Most advisers were faculty members of Department of Chemistry of University of Warsaw. The distribution of doctorates between those professors was very uneven. Most professors advised only one doctorate each, and the highest record was nine doctorates per professor (two cases). A correlation between the sex of the doctorate recipients on the one hand and of their Polish advisers on the other was observed. Among male Ph.D. candidates, 52 had male advisors, and only 7 had female advisors (ratio 7:1) while among female Ph.D. candidates, 61 had male advisors, and 33 had female advisors (ratio 2:1). The above numbers do not add up to 154, because one male Ph.D. candidate had two Polish advisers of opposite sexes. The present results indicate that male Ph.D. candidates had male advisors more frequently than female Ph.D. candidates. Similar gender correlation has been reported by Villarroya et al. (2008).

The following steps of scientific career of the advisors were analyzed: Master degree, Ph.D., habilitation, and current employment (when the doctorate was awarded). In six female advisers (11 % of all advisors) the information about the Master degree could not be confirmed in the available sources. This is probably because they received their Master degrees under their maiden names, which were different from their current names. Table 4 indicates that probably in almost ¾ of all advisers their entire career was connected with Department of Chemistry of University of Warsaw. Six other advisors received their Master degree at another university, but since doctorate their entire career was connected with Department of Chemistry of University of Warsaw. Six other advisors received their Master degree at Department of Chemistry of University of Warsaw, they received their Ph.D. elsewhere, but after doctorate their entire career was connected with their original Alma Mater. Only in six advisors who promoted 10 doctors, the career pattern was significantly different from a typical one.

**Table 4:** Characterization of Polish doctorate advisors

Property	% advisors	% doctorates
Entire career at Dept. Chem. Univ. Warsaw	63	61
Probably entire career at Dept. Chem. Univ. Warsaw	11	13
Entire career but Master at Dept. Chem. Univ. Warsaw	11	11
Entire career but Ph.D. at Dept. Chem. Univ. Warsaw	5	8
Other	11	6

Table 4 indicates that the academic inbreeding demonstrated in Table 2 for the scientists born about 1980, refers also the generation born about 1950 (professors).

159 professors participated in the doctorates as referees. The distribution of doctorates between those referees was very uneven. Most professors reviewed only one doctorate each, and the highest records were 14 and 13 doctorates per professor. Most multiple referees were faculty members of Department of Chemistry of University of Warsaw. The recent changes in the regulations in Poland will substantially change the fraction of internal referees, namely referees from the same university are not allowed any more.

## Discussion

Probably University of Warsaw plays a similar role among Polish universities as the Ivy League schools in the USA (Smyth and Mishra 2014) or Seoul National University in Korea (Anonymous 1998). The superiority of the University of Warsaw in chemistry is reflected in the average number of citations per paper. For example according to WoS^®^ it was 7 for all chemical papers with Polish affiliation published in 2010, and 11.35 for the chemical papers from University of Warsaw. Also the *h*-indices of the professors of chemistry from University of Warsaw (several professors with *h* > 40) are higher than the *h*-indices of their counterparts in most other Polish universities. The explanation “the best candidates happen to be our alumni” (Anonymous 1998) may explain a high level of academic inbreeding in the best schools. Such an attitude of the best schools induces high level of academic inbreeding in the other schools. Since the doors of the top universities are closed to alumni of doctoral studies from other universities, the less prestigious universities hire their own alumni to give them any chance of employment in the academic sector.

Information about doctor degrees of individuals is less frequently used in scientometric analyses than the data about scientific publications, because the data on individual doctorates is not easily available. Doctorates are underestimated as a source of scientometric data. For example, the data on individual doctorates provide information on field-to-field migrations of scientists, which cannot be derived from analysis of journal publications. An academic degree implies commitment to certain discipline, and that discipline may have serious consequences for further professional career of an individual. In contrast, publication of a paper in a journal, which belongs to different scientific discipline is not a real instance of migration. Therefore the present author is critical about studies of field-to-field migrations based solely on analysis of scientific publications.
